# Self-stigma and coping strategies in remitted Tunisian patients with bipolar disorder

**DOI:** 10.1192/j.eurpsy.2022.916

**Published:** 2022-09-01

**Authors:** R. Jenhani, S. Ellouze, D. Bougacha, F. Znaidi, R. Ghachem

**Affiliations:** 1 Razi hospital, Psychiatry B, Manouba, Tunisia; 2 Hedi Chaker University Hospital, Psychiatry B, Sfax, Tunisia; 3 Razi hospital, B, Manouba, Tunisia; 4 Razi Hospital, B, Manouba, Tunisia; 5 Razi Hospital, Psychiatry B, Manouba, Tunisia

**Keywords:** coping, bipolar disorder, self-stigma

## Abstract

**Introduction:**

Patients with bipolar disorder may adjust their behaviors and choose a coping strategy to face self-stigma and avoid unpleasant social and professional adversities. These coping orientations are either defensive, or active behavioral strategies.

**Objectives:**

The aim of this study was to assess self-stigma in remitted patients with bipolar disorder and to investigate coping strategies to struggle the internalized stigma.

**Methods:**

We conducted a cross-sectional, descriptive, and analytical study of 61 patients with bipolar disorder. Euthymia was verified using the Hamilton scale for depression and the Young scale for mania. We used the Internalized Stigma of Mental Illness (ISMI) to evaluate self-stigma, the Stigma coping orientation Scale (SCOS) to assess coping strategies.

**Results:**

The mean age of patients was 43.4 years. The sex ratio was 2.4. The mean score on the ISMI was 2.36. More than half of our patients (59%) were self-stigmatized. Secrecy (57%) and withdrawal (56%) were the most adopted coping strategies. The mean self-stigma score was significantly associated with higher scores on defensive coping strategies such as secrecy (p<10^-3^) and withdrawal (p<10^-3^). However, scores on challenging (p<10^-3^), education (p<10^-3^) and distancing (p=0.014) strategies were inversely correlated with self-stigma scores. The logistic regression analyses revealed a significant association between defensive coping strategies (secrecy and withdrawal) and internalized stigma.

**Conclusions:**

The relationship between defensive coping strategies and self-stigma appears to be bidirectional. Enhancing coping strategies oriented to education, challenging and engaging patients in social interaction and reducing the use of deleterious coping strategies focusing on secrecy and withdrawal may lead to restrict self-stigma.

**Disclosure:**

No significant relationships.

